# Interactive association between processing induced molecular structure changes and nutrient delivery on a molecular basis, revealed by cutting-edge vibrational biomolecular spectroscopy

**DOI:** 10.1186/s40104-019-0384-z

**Published:** 2019-10-22

**Authors:** Aya Ismael, Victor Hugo Guevara-Oquendo, Basim Refat, Peiqiang Yu

**Affiliations:** Ministry of Agriculture Strategic Research Chair (PY) Lab, Department of Animal and Poultry Science, College of Agriculture and Bioresources, University of Saskatchewan, 51 Campus Drive, Saskatoon, SK S7N 5A8 Canada

**Keywords:** Alpha-helix and beta-sheet, Amides (I and II), Biofunctional groups, Chemical structure, Protein metabolism and bioavailability, Protein molecular structure

## Abstract

**Background:**

This study was conducted to determine protein molecular structure profiles and quantify the relationship between protein structural features and protein metabolism and bioavailability of blend pelleted products (BPP) based on co-products (canola or carinata) from processing with different proportions of pulse pea screenings and lignosulfonate chemical compound.

**Method:**

The protein molecular structures were determined using the non-invasive advanced vibrational molecular spectroscopy (ATR-FT/IR) in terms of chemical structure and biofunctional groups of amides (I and II), α-helix and β-sheet.

**Results:**

The results showed that increasing the level of the co-products in BPP significantly increased the spectral intensity of the amide area and amide height. The products exhibited similar protein secondary α-helix to β-sheet ratio. The protein molecular structure profiles (amides I and II, α-helix to β-sheet) were highly associated with protein degradation kinetics and intestinal digestion. In conclusion, the non-invasive vibrational molecular spectroscopy (ATR-FT/IR) could be used to detect inherent structural make-up characteristics in BPP.

**Conclusion:**

The molecular structural features related to protein biopolymer were highly associated with protein utilization and metabolism.

**Electronic supplementary material:**

The online version of this article (10.1186/s40104-019-0384-z) contains supplementary material, which is available to authorized users.

## Background

Due to the high worldwide demands of oils and fuel in industries, bio-energy processing (bio-fuel, bio-oil, and bio-ethanol) resulted in huge amounts of co-products such as canola meal, new carinata meal, and distiller’s dried grains with solubles [1–3]. There are some studies that have investigated the application of the bio-coproduct of canola meal in ruminant or monogastric food/feed research [4–6]. Nevertheless, to our knowledge, there is limited information that could be found in the literature on the new bio-coproduct of carinata meal from bio-fuel processing when it is blended with other foodstuff such as peas screenings to optimize physicochemically or the biopolymer functions as well as nutritive value.

“Wet” chemistry analysis is usually used for feed evaluation, however, this technique usually damages the inherent structure of food/feed samples [7]. The biofunctions and nutritive value, as well as fermentation features, have been reported to be influenced by the different inherent molecular structure make-up and conformation [8]. The mid-infrared spectral region (ca. 4000–800 cm^−1^) has strong characteristic vibrational transitions compared with near-infrared region, especially in the wavelength range between ca. 1800 and 800 cm^−1^, which is called the “fingerprint region” [8, 9].

Vibrational spectroscopies such as Fourier transform infrared spectroscopy with attenuated total reflectance (ATR-FT/IR) is capable to detect the molecular structure of biomaterials. The ATR-FT/IR spectroscopy is a direct, rapid, non-destructive, and non-invasive bioanalytical technique used to reveal the infrared spectrum of absorptions or emissions of liquid, gas, or solids [10]. The ATR-FT/IR spectroscopy has advantages such as revealing the molecules structural changes of different types of food/feed and determining the nutrient utilization and bioavailability [11]. Moreover, this technique could recognize the molecular structure of different food crop varieties, food/feed ingredients, and studying the effects of food/feed processing on protein and carbohydrate-related molecular structures [4, 12–14]. However, there is no systematic study that has been conducted to determine how the blending process induces changes in protein intrinsic molecular structures and how these inherent structure changes influence protein metabolism and utilization. Therefore, the current study was performed to 1) investigate the differences among eight blend pellet products (BPP) from the bio-energy processing (new carinata meal vs. canola meal) with different proportions of pea screenings and lignosulfonate compound in terms of protein molecular structure; and 2) to quantify the protein inherent molecular structure changes in relation to protein profile, Cornell Net Carbohydrate, and Protein System (CNCPS) protein sub-fractions, energy values, protein digestion (rumen and intestine), and the metabolizable protein supply. The hypothesis of this study was that vibrational Fourier transform infrared spectroscopy could be used to detect an interactive association of processing induced molecule structural changes in biofunctional groups of protein amides I and II, alpha-helix and beta-sheet in the BPPs with protein metabolism and utilization.

## Methods

All experimental procedures used in this study were approved by the University of Saskatchewan Animal Care Committee (UCACS Protocol No. 19910012) and were conducted in accordance with the Canadian Council of Animal Care guidelines [15].

### Sample preparation for chemical and molecular structure studies

The experiment was performed at the SRP biomolecular spectroscopy lab, University of Saskatchewan (Saskatoon, Canada). The co-products of canola meal and new carinata meal from bio-fuel and bio-oil processing were used as BPPs by adding different levels of pulse co-products (pea screenings) and lignosulfonate compound. Eight blends were formulated; the BPP1 to BPP4 were based on new co-products of carinata meal (Agrisoma; Saskatoon, Canada) with different levels of pulse pea screenings and lignosulfonate; and the BPP5 to BPP8 were based on processing co-product of canola meal (Cargill Animal Nutrition, Clavet, Canada) with different levels of lignosulfonate and pulse pea screenings. The composition of the BPPs (on DM basis) is as follow: BPP1: lignosulfonate 0% + carinata meal 50% + pea screenings 50.0% DM. BPP2: lignosulfonate 4.8% + carinata meal 47.6% + pea screenings 47.6% DM. BPP3: lignosulfonate 0% + carinata meal 75% + pea screenings 25% DM. BPP4: lignosulfonate 4.8% + carinata meal 71.4% + pea screenings 23.8% DM. BPP5: lignosulfonate 0% + canola meal 50% + pea screenings 50.0% DM. BPP6: lignosulfonate 4.8% + canola meal 47.6% + pea screenings 47.6% DM. BPP7: lignosulfonate 0% + canola meal 75% + pea screenings 25% DM. BPP8: lignosulfonate 4.8% + canola meal 71.4% + pea screenings 23.8% DM. The use of different of the proportion of canola meal and pea screenings were made to obtain the best of amino acid profile and nutritional value. Adding lignosulfonate at 5%DM was based on a previous study [16] that showed a beneficial effect on canola meal by increasing the ruminal undegradable and the lactational performance of high producing dairy cows.

Pulse pea screenings were sourced from ILTA Grain Company (Surrey, Canada), while the lignosulfonate was obtained from Ameri-bond (Canada). The BPP was processed at two different times to make two different batches (*n* = 2) for each BPP. The blending and pelleting were conducted at Canadian Feed and Research Centre (CFRC, North Battleford, the University of Saskatchewan, Canada). For the pellet processing, the following procedure was followed to obtain the BPP: 1) mixing the combinations in the Scott Equipment model TSM 363 (New Prague, MN, USA) for 2 min.; 2) heating the different combinations by using Colorado Mill Equipment ECO-R30 (Cañon City, USA) at 65 °C and pelleting through a 3.6-mm diameter die such that the residence time of the blends in the die did not overtake 15 s; and 3) cooling at room temperature.

### Detection of blending and pelleting impact on biofunctions and nutritive value using wet chemistry and biological techniques

The detailed chemical compositions, nutrient profiles, degradation kinetic profile, intestinal digestion, and the true nutrients supply, as well as metabolizable protein and feed milk value, were reported previously by Guevara-Ouendo et al. [17]. The chemical profile, CNCPS fractions, and energy values of the BPPs (*n* = 16; carinata meal or canola meal with different combinations of pulse peas and lignosulfonate) are summarized in Table 1. These chemical and nutritive data were used for the correlation and regression studies on the association between the molecular structures and nutrition.

**Table 1:** Mean, standard deviation, minimum, and maximum values of basic chemical profile, protein subfractions and predicted energy profiles for combined feed of blend pelleted products with different combinations (two levels of lignosulfonate chemical compound, two types of co-products from biofuel (carinata) or bio-oil processing with two levels of each type, and two levels of pea screenings

Items	Mean (*n* = 16)	SD^a^	Minimum	Maximum
Basic chemical profile^b^				
CP, g/kg DM	392.2	38.0	336.3	457.9
NDICP, g/kg CP	99.2	37.4	55.5	150.8
ADICP, g/kg CP	23.2	9.2	10.6	36.8
SCP, g/kg CP	307.8	66.4	185.1	427.0
NPN, g/kg CP	32.75	4.15	24.66	41.41
Protein subfractions, CNCPS 6.5^c^				
PA2, g/kg CP	347.7	74.7	208.4	479.7
PB1, g/kg CP	553.1	53.1	464.8	655.4
PB2, g/kg CP	76.0	45.8	28.0	135.2
PC, g/kg CP	23.2	9.2	10.6	36.8
Predicted energy values by NRC^d^				
tdCP, g/kg DM	388.6	38.2	332.1	455.2
TDN_1×,_ g/kg DM	753.7	28.2	709.7	793.2
ME_3×,_ NRC–2001 dairy	2.98	0.12	2.80	3.15
NEL_3×,_ NRC–2001 dairy	1.91	0.09	1.78	2.03
Ruminal degradation kinetics of CP^e^				
Kd, %/h	91.7	23.8	53.9	178.9
S, g/kg	175.3	28.4	97.9	231.2
D, g/kg	708.2	40.6	620.4	774.3
U, g/kg	116.5	51	43.6	219.3
BCP, g/kg DM	403.3	60.8	320.1	523.5
EDCP, g/kg DM	596.7	60.8	476.5	679.9
Intestinal digestion of CP^f^				
dIDP, g/kg CP	735.6	54.4	630.7	833.7
IDP, g/kg	299.2	63.4	212.0	406.5
Total-tract digestion of CP^g^				
TDP, g/kg CP	895.9	13.2	862.1	929.3
Predicted values of potential nutrient supply to dairy cattle, g/kg DM^h^				
MCP_RDP_	197.91	19.45	6333.0	171.2
MCP_TDN_	89.95	3.30	2878.0	84.7
AMCP	57.57	2.11	1842.0	54.2
RUP	159.33	35.12	5099.0	111.6
ARUP	118.42	32.52	3789.0	79.5
MP	180.19	33.78	5766.0	140.3
DPB	126.70	25.49	4054.0	91.0
Feed milk value, kg milk/kg DM^i^				
FMV	3.39	0.78	108.3	2.1

^a^standard deviation

^b^CP, crude protein; NDICP, neutral detergent insoluble crude protein; ADICP, acid detergent insoluble crude protein; SCP, soluble crude protein; NPN, non-protein nitrogen

^c^PA2, soluble true protein; PB1, insoluble true protein. PB2, fiber-bound protein; PC, indigestible protein

^d^tdCP, truly digestible crude protein; TDN_1×_, total digestible nutrient at one times maintenance; ME_3×_, metabolizable energy at the production level of intake (3×); NEL_3×_, net energy for lactation at the production level of intake (3×)

^e^Kd, degradation rate; S, soluble fraction in the *in situ* incubation; D, potentially degradable fraction; U, undegradable fraction, BCP, bypass crude protein; EDCP, effectively degraded of crude protein

^f^dIDP, intestinal digestibility of rumen bypass protein on a percentage basis; IDP, intestinal digested crude protein,

^g^TDP, total digestion of crude protein

^h^MCP_RDP_, a microbial protein synthesized in the rumen based on available protein calculated as 0.85 of rumen degraded protein; MCP_TDN_, a microbial protein synthesized in the rumen based on available energy (discounted TDN); AMCP, truly absorbed rumen-synthesized microbial protein in the small intestine. RUP, ruminally undegraded feed CP, calculated according to the formula in NRC–2001 dairy model; ARUP, truly absorbed rumen–undegraded feed protein in the small intestine. MP, metabolizable protein (a true protein that is digested postruminally and the component amino acid absorbed by the intestine); DPB, reflects the difference between the potential microbial protein synthesis based on ruminally degraded feed CP and that based on energy-TDN available for microbial fermentation in the rumen

^i^FMV, feed milk value

The CP was analyzed according to AOAC official method 984.13 [18]. The protein subfractions i.e. neutral detergent insoluble crude protein (NDICP), non–protein nitrogen (NPN), and acid detergent insoluble crude protein (ADICP) were determined using the methods described by [19]. The soluble crude protein (SCP) was analyzed by incubating samples with bicarbonate-phosphate buffer then filtrating through Whatman filter paper [20]. For energy profiles, total digestible nutrient (TDN), metabolizable energy (ME), digestible energy (DE), and net energy (NE) were used for estimating the available energy in BPP. The truly digestible crude protein (tdCP), total digestible nutrient at a maintenance level (TDN_1×_), digestible energy at a production level of intake (DE_3×_), metabolizable energy at a production level of intake (ME_3×_), net energy for lactation at a production level (NEL_3×_) were determined by using a summative approach of the NRC [21].

The *in situ* degradation kinetics and the intestinal digestion of CP were performed according to [22]. Degradation characteristics of CP were determined by applying the first-order kinetic model described by [23]. The results were calculated using the NLIN procedure of SAS 9.4 and iterative least-squares regression (Gausse Newton method):

$$ \mathrm{R}\left(\mathrm{t}\right)=\mathrm{U}+\mathrm{D}\times {\mathrm{e}}^{-\mathrm{Kd}\times \left(\mathrm{t}-\mathrm{T}0\right)} $$

where U is the undegradable fraction (%); D is the potentially degradable fraction (%); Kd is the degradation rate (%/h), and T0 is the lag time (h).

The bypass crude protein (BCP) was determined according to the NRC model:

$$ \%\mathrm{BCP}=\mathrm{U}+\mathrm{D}\times \mathrm{Kp}/\left(\mathrm{Kp}+\mathrm{Kd}\right) $$

The truly nutrient supply was estimated using the NRC model. In this model, the MP (g/kg DM) is calculated based on the following equation [21]:

$$ \mathrm{MP}=\mathrm{AMCP}+\mathrm{ARUP}+\mathrm{AECP} $$

where ME is the metabolizable protein; AMCP is the absorbable microbial protein; ARUP is the truly absorbable rumen undegraded feed protein, and AECP is the truly absorbable endogenous protein in the small intestine.

The milk value (FMV) was predicted based on the metabolizable protein content of BPP, where the efficiency of utilizing of metabolizable protein is assumed to be 0.67 and protein composition in milk is assumed to be 33 g protein/kg of milk [21].

### Detection of blending and pelleting impact on protein molecular structure changes and collecting molecular spectra related to the protein primary and secondary structural components

For the molecular spectral analysis, the samples were grounded to pass a 0.12-mm sieve (Retsch ZM200, Rose, Scientific Ltd., Canada) for ATR-FT/IR spectroscopic analysis. Five subsamples from every sample were spectroscopically scanned. The molecular spectral data of samples were collected and corrected for the background spectrum using ATR-FT/IR molecular spectroscopy (JASCO 4200, JASCO International Co. Ltd., Tokyo, Japan). The spectra were generated in the mid-IR (ca. 4000–800 cm^−1^; Additional file 1: Figure S1) and the fingerprint region (ca. 1800–800 cm^−1^) with a spectral resolution of 4 cm^−1^. The ATR- FT/IR spectral was processed by using OMNIC 7.3 (Spectra-Tech, Madison, WI). The regions of specific interest in this study included the primary molecular protein structural (amide I and amide II) and the secondary molecular protein structural (α-helix and β-sheet) in the mid-IR. The structural spectral features on the protein were determined by analyzing the absorption peak parameter such as spectral region, baseline, peak, height, and area according to the published methods [8].

**Fig. 1:**
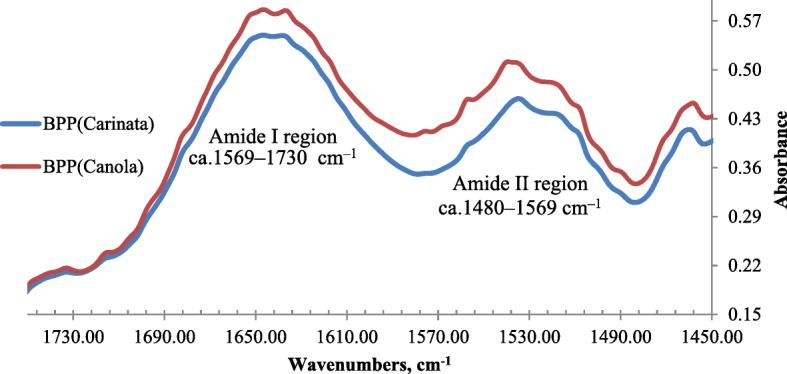
Typical Fourier transform infrared attenuated total reflectance (FT/IR-ATR) spectrum of the blend pelleted products (BPP) based on carinata with pea screenings or canola meal with pea screenings for protein region ca. 1730 to 1480 cm^−1^, showing the functional makeup of protein amide I and II

The univariate spectral analyses of protein structure comprised the primary and the secondary protein structures. The primary protein structures included amide I and II. The baseline of protein spectral was centered at ca. 1480–1730 cm^−1^ (Fig. 1). The baseline of the amide I area was centered at ca. 1569–1730 cm^−1^. The baseline of the amide II area was centered at ca. 1480–1569 cm^−1^. The peak height of the amide I was centered at ca. 1638–1649 cm^−1^, while the peak height of amide II was centered at ca. 1533–1540 cm^−1^. The secondary protein structures of the amide I region were determined by using the 2^nd^ Derivative Function and Fourier Self–Deconvolution function in OMNIC 7.4 Software (Spectra Tech, Madison, WI) according to published methods [24, 25]. The secondary protein structures mainly comprised α-helix and β-sheet. The peak height of α-helix was centered at ca. 1647–1653 cm^−1^, while the peak height of β-sheet was centered at ca. 1625–1631 cm^−1^.

Principal Component Analysis (PCA) was performed using the Unscrambler 10.3 (CAMO Software AS, Oslo, Norway) for clustering any variation among BPP products. The raw data were prepossessed using baseline offset. The Savitzky-Golay algorithm was used to calculate the second derivative. Principal components with high eigenvalues were selected for further analysis. Two-dimensional score plots were used to display the PCA scores among the treatments. Loading plots for the important principal components were used to display the relations among PC components with IR variable data.

### Statistical analysis

The data of functional groups in the protein region (ca. 1480–1730 cm^−1^) were analyzed by SAS 9.4 (SAS Institute, Inc., Cary, NC, USA). The experiment was designed using the randomized complete block design (RCBD) with blending/pellet processing time run as a random block effect.

The correlation between the biofunctional groups related to protein region (amide I, II peak highest and areas, α-helix, β-sheet and their ratio) and the chemical profiles of protein, energy values, degradation kinetics parameters, intestinal digestive characteristics of protein, and the truly absorbed protein supply was analyzed by using the PROC CORR procedure in SAS 9.4 (SAS Institute, Inc., Cary, NC, USA). Rank correlation with the SPEARMAN option and normality test with the UNIVARIATE option was used in the correlation study.

Multiple regression analysis (with model variable selection method) was performed to select the best biofunctional groups that would explain the nutritive values of BPP using the PROC REG procedure of SAS with a reversed stepwise option. The following model where be used for the multiple regression with model variable selection: model Y = spectral parameter 1 + spectral parameter 2 + spectral parameter 3 + spectral parameter 4 + ... + error. The model used a “STEPWISE” option with variable selection criteria: “SLENTRY = 0.05, SLSTAY = 0.05”. All variables left in the final prediction models were significant at the 0.05 level. Residual analysis was performed using the Univariate procedure of SAS with Normal and Plot options. Collinearity detection was conducted using the VIF option of SAS to eliminate the influence of correlated dependent variables to the prediction of independent variables.

## Results

### Effect of blending/pelleting on protein molecular spectral intensities in blend pelleted products

The protein molecular spectral profiles include the primary and secondary structures of protein of different BPPs by using ATR-FT/IR vibrational spectroscopy is shown in Table 2. The results showed that BPP7 and BPP3 had the highest (*P* < 0.01) amide I peak height (averaged 0.307 IU), while the BPP2 and BPP6 had the lowest values (averaged 0.259 IU). The amide I area was significantly different (*P* < 0.01) among BPPs, where the BPP7 and BPP3 had the highest amide I area (averaged 21.2 IU), whereas the BPP2 and BPP6 had the lowest values.

**Table 2.** Protein spectral profile (protein primary structures and protein secondary structures) of different blend pelleted products (BPP) of carinata meal and canola meal in the different levels of pea screenings and lignosulfonate and comparison between carinata meal and canola meal (CR vs. CN), adding lignosulfonate with no adding lignosulfonate (LSO_3_ No vs. Add), and high level of pea screenings with low level of pea screenings (Pea High vs. Low) using FTIR Vibrational Spectroscopy

CO-P	Pea	LSO_3_	Treatments^1^	Amide I peak height^4^	Amide II peak height	Amide I, II height ratio	Amide I area	Amide II area	Amide area	α-helix height	β-sheet height	α-helix, β-sheet height ratio
CR	High	NO	BPP1 (*n* =2)	0.279^cd^	0.134^bc^	2.067^bc^	19.024^d^	6.469^bc^	25.494^de^	0.319^cd^	0.274^de^	1.166
CR	High	Add	BPP2 (*n* =2)	0.252^e^	0.123^d^	2.028^bc^	17.507^e^	5.760^d^	23.269^f^	0.285^e^	0.245^f^	1.167
CR	Low	NO	BPP3 (n =2)	0.302^ab^	0.140^b^	2.152^ab^	21.253^a^	6.532^b^	27.784^ab^	0.341^b^	0.299^bc^	1.141
CR	Low	Add	BPP4 (*n* =2)	0.281^c^	0.126^cd^	2.218^a^	20.008^c^	6.008^cd^	26.017^cd^	0.319^cd^	0.281^cde^	1.137
CN	High	NO	BPP5 (*n* =2)	0.279^cd^	0.143^b^	1.957^cd^	19.032^d^	6.408^bc^	25.441^de^	0.331^bc^	0.288^cd^	1.151
CN	High	Add	BPP6 (*n* =2)	0.265^de^	0.140^b^	1.934^cd^	18.008^e^	6.554^b^	24.562^e^	0.313^d^	0.266^e^	1.185
CN	Low	NO	BPP7 (*n* =2)	0.312^a^	0.164^a^	1.892^d^	21.090^ab^	7.642^a^	28.732^a^	0.374^a^	0.334^a^	1.120
CN	Low	Add	BPP8 (*n* =2)	0.288^bc^	0.138^b^	2.139^ab^	20.225^bc^	6.605^b^	26.827^bc^	0.345^b^	0.311^b^	1.110
SEM^2^				0.0058	0.0041	0.0692	0.3357	0.2136	0.429	0.0067	0.0073	0.0201
*P* value				<0.01	<0.01	<0.01	<0.01	<0.01	<0.01	<0.01	<0.01	0.18
Contrasts *P* value^3^	CO-P:	CR vs. CN		0.04	<0.01	<0.01	0.52	<0.01	0.02	<0.01	<0.01	0.45
	LSO_3_:	No vs. Add		<0.01	<0.01	0.07	<0.01	<0.01	<0.01	<0.01	<0.01	0.67
	Pea:	High vs. Low		<0.01	<0.01	<0.01	<0.01	<0.01	<0.01	<0.01	<0.01	<0.01

^a-f^Means with the different letters in the same column are significantly different (*P* < 0.05)

^1^BPP: blend pelleted product; BPP1: lignosulfonate 0% DM + carinata meal 50% DM + pea screenings 50.0% DM.; BPP2: lignosulfonate 4.8% DM + carinata meal 47.6% DM + pea screenings 47.6% DM; BPP3: lignosulfonate 0 % DM + carinata meal 75 % DM + pea screenings 25% DM; BPP4: lignosulfonate 4.8% DM + carinata meal 71.4% DM + pea screenings 23.8% DM; BPP5: lignosulfonate 0% DM + canola meal 50% DM + pea screenings 50.0% DM; BPP6: lignosulfonate 4.8% DM + canola meal 47.6% DM + pea screenings 47.6% DM; BPP7: lignosulfonate 0% DM + canola meal 75% DM + pea screenings 25% DM; BPP8: lignosulfonate 4.8% DM + canola meal 71.4% DM + pea screenings 23.8% DM

^2^SEM: Standard error of means

^3^CO-P: Co-Product. CR: Carinata meal. CN: Canola meal. LSO_3_: Lignosulfonate

^4^Baseline for protein spectral peak: ca. 1480–1730 cm^–1^; protein amide I region: ca. 1569–1730 cm^–1^; protein amide II region: ca. 1480–1569 cm^–1^; center range of amide I peak: ca. 1638–1649 cm^–1^; center range of amide II peak: ca. 1533–1540 cm^–1^; center range for α–helix: ca. 1647–1653cm^–1^; center range for β-sheet: ca. 1625–1631cm^–1^

Our results showed that the ratio of amide I to amide II was higher (*P* < 0.05) in BPP based on the new co-product of carinata meal compared with co-product of canola meal (2.12 vs. 1.98). Furthermore, adding the pulse pea screenings decreased (*P* < 0.05) the ratio of amide I to amide II in BPP based on the new co-product of carinata meal from 2.19 to 2.05, and in BPP based on the co-product of canola from 1.95 to 2.02.

The secondary structures such as α-helix and β-sheet and their ratio of BBPs are presented in Table 2. It has been found that the α-helix, β-sheet height ratio was the same for all treatments. This study showed that the ratio of α-helix to β-sheet decreased (*P* < 0.05) from 1.17 to 1.13 by decreasing the level of co-products in the BPPs.

### Correlation analysis between protein molecular spectral features and nutrition profiles in the blend pelleted products

The correlation analysis between the vibrational spectral features and protein profiles, protein subfractions and the energy values of BPPs are shown in Table 3. The CP had positive correlations with amide I area (*r* = 0.70, *P* < 0.05), total amide area (*r* = 0.58, *P* = 0.02), and amide I height (*r* = 0.55, *P* = 0.03) in BPPs. However, CP exhibited a negative correlation with the ratio of α-helix to β-sheet (*r* = − 0.55, *P* = 0.03).

**Table 3:** Correlation between chemical nutrient composition and protein fractions of blend pelleted products with different combinations (*n* = 16) (two levels of lignosulfonate chemical compound, two types of co-products from biofuel (carinata meal) and-oil processing (canola meal) with two levels of each type, and two levels of pea screenings and molecular structure related to amide region

Items		Amide I height	Amide II height	Amide I, II ratio	Amide I area	Amide II area	Amide area	α-helix height	βsheet height	α-helix, *β*-sheet ratio
Basic protein profile^a^										
CP, g/kg DM	*r*	0.55	0.11	0.40	0.70	0.16	0.58	0.39	0.44	− 0.55
	*P* value	0.03	0.69	0.13	0.01	0.56	0.02	0.14	0.08	0.03
NDICP, g/kg CP	*r*	− 0.06	− 0.50	0.52	0.08	−0.40	− 0.08	− 0.33	− 0.32	0.12
	*P* value	0.82	0.05	0.04	0.77	0.13	0.77	0.21	0.23	0.66
ADICP, g/kg CP	*r*	0.29	0.56	−0.36	0.18	0.51	0.32	0.53	0.55	−0.40
	*P* value	0.27	0.02	0.17	0.49	0.04	0.23	0.03	0.03	0.13
SCP, g/kg CP	*r*	0.25	0.48	−0.38	0.05	0.35	0.16	0.41	0.37	−0.12
	*P* value	0.36	0.06	0.15	0.85	0.19	0.56	0.12	0.15	0.65
NPN, g/kg CP	*r*	−0.71	−0.12	−0.56	−0.80	− 0.22	−0.68	− 0.61	−0.67	0.64
	*P* value	0.01	0.67	0.02	0.01	0.42	0.01	0.01	0.01	0.01
Predicted total digestible nutrients, g/kg DM^b^										
tdCP	*r*	0.53	0.09	0.41	0.68	0.13	0.56	0.36	0.42	−0.53
	*P* value	0.03	0.75	0.12	0.01	0.62	0.02	0.17	0.11	0.03
TDN_1×_	*r*	−0.38	− 0.53	0.23	−0.33	− 0.50	− 0.43	−0.59	− 0.63	0.55
	*P* value	0.15	0.03	0.40	0.21	0.05	0.10	0.02	0.01	0.03
ME_3×_	*r*	−0.12	− 0.43	0.36	− 0.02	− 0.38	− 0.15	− 0.38	−0.39	0.27
	*P* value	0.67	0.09	0.17	0.94	0.14	0.58	0.15	0.13	0.30
NEL_3×_	*r*	−0.12	−0.44	0.37	−0.02	− 0.39	− 0.15	− 0.37	−0.39	0.27
	*P* value	0.67	0.09	0.16	0.95	0.14	0.59	0.15	0.14	0.32
CNCPS protein subfractions, g/kg CP^c^										
PA2	*r*	0.23	0.47	−0.39	0.04	0.34	0.15	0.40	0.36	−0.10
	*P* value	0.39	0.06	0.13	0.90	0.19	0.59	0.13	0.17	0.71
PB1	*r*	−0.28	− 0.31	0.18	− 0.10	− 0.20	− 0.15	− 0.32	−0.28	0.06
	*P* value	0.29	0.24	0.50	0.70	0.46	0.58	0.22	0.29	0.83
PB2	*r*	−0.11	−0.52	0.50	0.03	−0.43	− 0.13	− 0.38	−0.37	0.18
	*P* value	0.68	0.04	0.05	0.92	0.10	0.64	0.15	0.16	0.51
PC	*r*	0.29	0.56	−0.36	0.18	0.51	0.32	0.53	0.55	−0.40
	*P* value	0.27	0.02	0.17	0.49	0.04	0.23	0.03	0.03	0.13

^a^CP, crude protein; NDICP, neutral detergent insoluble crude protein; ADICP, acid detergent insoluble crude protein; SCP, soluble crude protein; NPN, non-protein nitrogen

^b^tdCP, truly digestible crude protein; TDN_1×_, total digestible nutrient at one times maintenance; ME_3×_, metabolizable energy at the production level of intake (3×); NEL_3×_, net energy for lactation at production level of intake (3×)

^c^PA2, soluble true protein; PB1, insoluble true protein. PB2, fiber-bound protein; PC, indigestible protein

The results in the current study showed NDICP had a negative correlation with amide II height (*r* = − 0.50, *P* = 0.05) and a positive correlation with the ratio of amide I to amide II height (*r* = 0.52; *P* = 0.04). The content of ADICP was found to be positively correlated with the amide II height (*r* = 0.56, *P* = 0.02) or the amide II area (*r* = 0.51, *P* = 0.04). The concentration of NPN was negatively correlated with the amide I area (*r* = − 0.80, *P* = 0.01), amide I height (*r* = − 0.71, *P* < 0.05), amide I to amide II area ratio (*r* = − 0.68, *P* < 0.05), amide I to amide II height ratio (*r* = − 0.56, *P* < 0.05), β-sheet height (*r* = − 0.67, *P* < 0.05), and α-helix height (*r* = − 0.61, *P* < 0.05). However, there was a positive correlation between NPN and the ratio of α-helix to β-sheet (*r* = 0.64, *P* = 0.01).

For the truly digestible crude protein, the results showed correlations between tdCP and amide I area (*r* = 0.68, *P* = 0.01), amide I height (*r* = 0.53, *P* < 0.05), the amide area (*r* = 0.56, *P* = 0.02). There was no correlation (*P* > 0.05) between the energy values and the molecular structure features related to protein region.

For protein sub fractions partitioned by the CNCPS model, the results showed that the slowly degradable protein (PB2 fraction) was positively associated with the amide I to amide II height ratio (*r* = 0.50, *P* = 0.05) and negatively related to the amide II height (*r* = − 0.52, *P* = 0.04). The PC fraction was observed to be positively associated with the amide II height (*r* = 0.56, *P* = 0.02) and amide II area (*r* = 0.51, *P* = 0.04).

In the current study, it has been found that the protein degradation rate was negatively correlated with the amide I area (*r* = − 0.36, *P* < 0.01) and the amide I to amide II height ratio (*r* = − 0.36, *P* = 0.04), while the slowly degradable fraction of protein was positively correlated with the amide I height, amide I area and amide area. The amide II area and the ratio of amide I to amide II height were found to be negatively related to the EDCP of BPPs (*P* < 0.05; Table 4). Additionally, the undegradable CP fraction of BPPs was found to be negatively correlated with the heights or areas of amide I and amide II. The *in vitro* digestion of BCP (% dIDP) and the total intestinal digestibility of CP (IADP %) have been found to be positively correlated with the amide I to amide II height ratio (*r* = 0.55, *P* < 0.05). The results showed that the α-helix to β-sheet ratio was correlated with the slowly degradable fraction of CP (*r* = − 0.44, *P* = 0.01) and the undegradable fraction of CP (*r* = 0.45, *P* = 0.01).

**Table 4:** Correlation between molecular structure related to amide region and predicted protein supply form combined feed (BPP; carinata meal and canola meal) using DVE system

Items		Amide I height	Amide II height	Amide I, II ratio	Amide I area	Amide II area	Amide area	α-helix height	β-sheet height	α-helix, β-sheet ratio
*In situ* ruminal degradation of CP^a^										
Kd, %/h	*r*	− 0.22	0.13	−0.53	− 0.36	0.12	− 0.19	0.02	− 0.09	0.25
	*P* value	0.22	0.49	< 0.01	0.04	0.53	0.30	0.90	0.62	0.17
S, g/kg	*r*	0.23	0.67	−0.65	−0.03	0.59	0.18	0.44	0.32	−0.10
	*P* value	0.20	< 0.01	< 0.01	0.85	< 0.01	0.33	0.01	0.07	0.59
D, g/kg	*r*	0.35	0.24	0.12	0.40	0.25	0.38	0.44	0.47	−0.44
	*P* value	0.05	0.18	0.50	0.02	0.17	0.03	0.01	< 0.01	0.01
U, g/kg	*r*	−0.40	−0.57	0.32	−0.25	−0.50	− 0.36	−0.60	− 0.56	0.45
	*P* value	0.02	< 0.01	0.07	0.16	< 0.01	0.04	< 0.01	< 0.01	< 0.01
BCP, g/ kg DM	*r*	0.13	−0.35	0.58	0.34	−0.29	0.15	−0.17	− 0.03	− 0.14
	*P* value	0.46	0.05	< 0.01	0.06	0.11	0.40	0.36	0.88	0.45
EDCP, g/ kg DM	*r*	0.65	0.62	−0.20	0.58	0.53	0.68	0.75	0.77	−0.64
	*P* value	< 0.01	< 0.01	0.26	< 0.01	< 0.01	< 0.01	< 0.01	< 0.01	< 0.01
*In vitro* intestinal digestion of CP^b^										
dIDP, g/kg CP	*r*	−0.16	−0.58	0.55	−0.04	− 0.47	− 0.19	− 0.39	−0.38	0.16
	*P* value	0.39	< 0.01	< 0.01	0.85	0.01	0.30	0.03	0.03	0.38
IDP, g/kg	*r*	0.18	−0.34	0.61	0.36	− 0.27	0.18	− 0.12	0.01	− 0.13
	*P* value	0.31	0.06	< 0.01	0.04	0.13	0.33	0.53	0.97	0.47
Total tract digestibility of CP^c^										
TDP, g/kg	*r*	0.02	−0.18	0.27	−0.02	−0.14	− 0.10	0.01	− 0.04	0.18
	*P* value	0.91	0.33	0.13	0.91	0.45	0.58	0.95	0.81	0.31
TDP, %	*r*	0.61	0.09	0.43	0.74	0.10	0.62	0.37	0.51	−0.54
	*P* value	< 0.01	0.61	0.01	< 0.01	0.60	< 0.01	0.04	< 0.01	< 0.01

^a^Kd, degradation rate; S, soluble fraction in the *in situ* incubation; D, potentially degradable fraction; U, undegradable fraction, BCP, bypass crude protein; EDCP, effectively degraded of crude protein

^b^dIDP, intestinal digestibility of rumen bypass protein on a percentage basis; IDP, intestinal digested crude protein

^c^TDP, total digestion of crude protein

The correlation between protein molecular structure and the truly absorbable protein supply of BPP is shown in Table 5. The data showed significant correlation between AMCP and the amide II height (*r* = − 0.53, *P* < 0.01), the amide II area (*r* = − 0.50, *P* < 0.01), the amide area (*r* = − 0.43, *P* < 0.01), and the helix to β-sheet ratio (*r* = 0.55, *P* < 0.01). For the truly absorbable rumen undegraded protein in the small intestine, ARUP has exhibited a positive correlation with the amide I to amide II height ratio (*r* = 0.62, *P* < 0.01). The MP had a positive correlation with amide I to amide II height ratio (*r* = 0.61, *P* < 0.01) and a negative correlation with the amide II height (*r* = − 0.43, *P* = 0.02). The DPB had positive correlations with amide II height (*r* = 0.70, *P* < 0.01), the total amide area (*r* = 0.65, *P* < 0.01), amide II area (*r* = 0.64, *P* < 0.01), amide I height (*r* = 0.63, *P* < 0.01), amide I area (*r* = 0.57, *P* < 0.01), β-sheet height (*r* = 0.79, *P* < 0.01), and α-helix height (*r* = 0.77, *P* < 0.01) but a negative correlation with α-helix to β-sheet ratio (*r* = − 0.62, *P* < 0.01). The FMV had a positive correlation with amide I to amide II height ratio (*r* = 0.45, *P* = 0.01), while negatively correlated with the amide II height (*r* = − 0.39, *P* = 0.03).

**Table 5:** Correlation between molecular structure related to amide region and predicted protein supply form combined feed (BPP; carinata meal and canola meal) using NRC system

Items		Amide I height	Amide II height	Amide I, II ratio	Amide I area	Amide II area	Amide area	α-helix height	β-sheet height	α-helix, β-sheet ratio
Absorbable microbial protein synthesis in the rumen, AMCP^a^										
MCP_RDP_	*r*	0.64	0.68	−0.25	0.58	0.62	0.65	0.76	0.78	−0.60
	*P* value	< 0.01	< 0.01	0.16	< 0.01	< 0.01	< 0.01	< 0.01	< 0.01	< 0.01
MCP_TDN_	*r*	−0.38	−0.53	0.23	−0.33	− 0.50	− 0.43	− 0.59	−0.63	0.55
	*P* value	0.03	< 0.01	0.22	0.06	< 0.01	0.02	< 0.01	< 0.01	< 0.01
AMCP	*r*	−0.38	−0.53	0.23	−0.33	− 0.50	− 0.43	− 0.59	−0.63	0.55
	*P* value	0.03	< 0.01	0.22	0.06	< 0.01	0.02	< 0.01	< 0.01	< 0.01
Truly absorbable rumen–the undegraded protein in the small intestine, ARUP^b^										
RUP	*r*	0.17	−0.33	0.59	0.37	−0.24	0.19	−0.08	− 0.03	− 0.20
	*P* value	0.35	0.07	< 0.01	0.04	0.19	0.29	0.66	0.86	0.27
ARUP	*r*	0.11	−0.41	0.62	0.30	−0.31	0.12	−0.15	− 0.11	−0.12
	*P* value	0.54	0.02	< 0.01	0.09	0.09	0.50	0.41	0.55	0.50
Total metabolizable protein (MP) and degraded protein balance, DPB^c^										
MP	*r*	0.08	−0.43	0.61	0.27	− 0.32	0.09	− 0.18	− 0.15	−0.09
	*P* value	0.65	0.02	< 0.01	0.14	0.07	0.62	0.32	0.43	0.64
DPB	*r*	0.63	0.70	−0.26	0.57	0.64	0.65	0.77	0.79	−0.62
	*P* value	< 0.01	< 0.01	0.15	< 0.01	< 0.01	< 0.01	< 0.01	< 0.01	< 0.01
Feed milk values based on metabolic characteristics of protein predicted by NRC system^d^										
FMV	*r*	−0.02	−0.39	0.45	0.15	−0.30	0.01	−0.25	−0.23	0.02
	*P* value	0.91	0.03	0.01	0.43	0.10	0.97	0.17	0.21	0.90

^a^MCP_RDP_, a microbial protein synthesized in the rumen based on available protein calculated as 0.85 of rumen-degraded protein; MCP_TDN_, a microbial protein synthesized in the rumen based on available energy (discounted TDN); AMCP, truly absorbed rumen-synthesized microbial protein in the small intestine

^b^RUP, ruminally undegraded feed CP, calculated according to the formula in NRC–2001 dairy model; ARUP, truly absorbed rumen–undegraded feed protein in the small intestine

^c^MP, metabolizable protein (a true protein that is digested postruminally and the component amino acid absorbed by the intestine); DPB, reflects the difference between the potential microbial protein synthesis based on ruminally degraded feed CP and that based on energy-TDN available for microbial fermentation in the rumen

^d^FMV, feed milk value

### Model variable selection analysis to choose the most important protein spectral parameters to predict the protein nutrient profile and protein utilization and metabolism

The multiple regressions analysis is shown in Table 6. The equations of protein profiles showed that CP could be predicted from amide I area and α-helix height, taking 79% of the total variance. The SCP could be predicted from amide I to amide II ratio area and α-helix height and 68% of the total variance was taken by it. Amide I to amide II ratio could also predict NDICP and NPN. The amide II height could be used to predict the ADICP, PB2, and PC. For truly digestible nutrients, tdCP could be predicted from amide I area and α-helix height, while the Amide I height and β-sheet height could predict TDN_3×_ with a total variance of 82%.

**Table 6:** Multiple regression analysis to choose the most important spectral parameters to predict protein profile and energy profile

Predicted variable, Y	Variable selection (variables left in the model with *P* < 0.05)	Equation prediction: Y = a + b_1_ × *x*_1_ + b_2_ × *x*_2_ + … … .	Model R^2^	RSD^a^	*P* value
Basic protein profile^b^					
CP, g/kg DM	Amide I area, α-helix height	Y = 4.19 + 4.12 × Amide I area – 137.95 × α-helix height	0.73	2.048	< 0.01
NDICP, g/kg CP	Amide I, II ratio	Y = −17.26 + 13.27 × Amide I, II ratio	0.27	3.304	0.04
ADICP, g/kg CP	Amide II height	Y = −2.65 + 35.84 × Amide II height	0.32	0.792	0.02
NPN, g/kg CP	Amide I, II ratio, Amide I area	Y = 93.01–10.83 × Amide I, II ratio – 1.95 × Amide I area	0.78	2.081	< 0.01
Predicted energy values by NRC, 2001^c^					
tdCP, g/kg DM	Amide I area, α-helix height	Y = 4.47 + 4.21 × Amide I area – 145.52 × α-helix height	0.73	2.137	< 0.01
TDN_1×_, g/kg DM	Amide I height, β-sheet height	Y = 72.96 + 241.16 × Amide I height – 228.54 × β-sheet height	0.81	1.301	< 0.01
Protein subfractions, CNCPS 6.5^d^					
PB2, g/kg CP	Amide II height	Y = 30.35–164.28 × Amide II height	0.27	4.053	0.04
PC, g/kg CP	Amide II height	Y = −2.65 + 35.84 × Amide II height	0.32	0.792	0.02

^a^RSD, residual standard deviation

^b^CP, crude protein; NDICP, neutral detergent insoluble crude protein; ADICP, acid detergent insoluble crude protein; NPN, non-protein nitrogen

^c^tdCP, truly digestible crude protein; TDN_1×_, total digestible nutrient at one times maintenance

^d^PB2, fiber-bound protein; PC, indigestible protein

Table 7 shows that the amide I area and the α-helix height could be used to estimate the Kd and the undegradable fraction of CP with 46% and 62% of the total variance, respectively. The amide I height and β-sheet height were the best spectral variables to predict the EDCP with 67% of the variance. The amide I, II height ratio, Amide area, β-sheet height and α-helix to β-sheet ratio could be used to predict the intestinal digestion of CP with 90% of the variance. The results in Table 8 shows that the amide I to amide II height ratio was the best spectral feature in estimating the ARUP, MP, and FMV of BPPs, while the amide I height and β-sheet height would be used to estimate the DBP and AMCP.

**Table 7:** Multiple regression analysis to choose the most important protein spectral parameters to predict protein ruminal digestion of CP

Predicted variable, Y	Variable selection (variables left in the model with *P* < 0.05)	Equation prediction: Y = a + b_1_ × *x*_1_ + b_2_ × *x*_2_ + … … .	Model R^2^	RSD^a^	*P* value
Degradation kinetics of CP^b^					
Kd, %/h	Amide I area, α-helix height	Y = 21.70–2.33 × Amide I area – 100.29 × α-helix height	0.46	1.808	< 0.01
S, g/kg	Amide II height, Amide II area	Y = 1.57 + 399.24 × Amide II height – 6.05 × Amide II area	0.51	2.050	< 0.01
D, g/kg	Amide I height, β-sheet height	Y = 65.67–199.00 × Amide I height + 213.44 × β-sheet height	0.42	3.202	< 0.01
U, g/kg	Amide area, α-helix height	Y = 30.82 + 3.87 × Amide area − 364.64 × α-helix height	0.62	3.252	< 0.01
BCP, g/kg DM	Amide I, II ratio	Y = − 132.49 + 14,247 × Amide I, II ratio	0.34	28.925	< 0.01
EDCP, g/kg DM	Amide I height, β-sheet height	Y = 125.35–827.29 × Amide I height – 1187.03 × β-sheet height	0.67	13.537	< 0.01
Intestinal digestibility of CP^c^					
dIDP, g/kg CP	Amide II height, Amide II area	Y = 102.68–749.79× Amide II height + 11.50 × Amide II area	0.48	4.055	< 0.01
IDP, g/kg	Amide I, II ratio, Amide area, β-sheet height, α, β- ratio	Y = 113.54 + 19.77 × Amide I, II ratio + 6.73 × Amide area − 571.92 × β-sheet height–117.54 × α, β- ratio	0.90	2.149	< 0.01
Total tract– digestibility of CP^d^					
TDP, g/kg DM	Amide I area, α-helix height	Y = 42.01 + 37.43 × Amide I area – 1282.98 × α-helix height	0.76	16.705	< 0.01

^a^RSD, residual standard deviation

^b^Kd, degradation rate; S, soluble fraction in the *in situ* incubation; D, potentially degradable fraction; U, undegradable fraction, BCP, bypass crude protein; EDCP, effectively degraded of crude protein

^c^dIDP, intestinal digestibility of rumen bypass protein on a percentage basis; IDP, intestinal digested crude protein,

^d^TDP, total digestion of crude protein

**Table 8:** Multiple regression analysis to choose the most important protein spectral parameters to predict protein supply using the NRC model

Predicted variable, Y	Variable selection (variables left in the model with *P* < 0.05)	Equation prediction: Y = a + b_1_ × *x*_1_ + b_2_ × *x*_2_ + … … .	Model R^2^	RSD^a^	*P* value
Absorbable microbial protein synthesis in the rumen (AMCP)^b^					
MCP_RDP_	Amide I height, β-sheet height	Y = 106.55–703.13 × Amide I height + 1008.90 × β-sheet height	0.67	11.507	< 0.01
MCP_TDN_	Amide I height, β-sheet height	Y = 87.07 + 287.78 × Amide I height – 272.73 × β-sheet height	0.82	1.470	< 0.01
AMCP	Amide I height, β-sheet height	Y = 55.72 + 184.17 × Amide I height – 174.54 × β-sheet height	0.82	0.941	< 0.01
Truly absorbable rumen-the undegraded protein in the small intestine (ARUP)^c^					
RUP	Amide I, II ratio	Y = −132.49 – 142.47 × Amide I, II ratio	0.34	28.926	< 0.01
ARUP	Amide I, II ratio	Y = −165.71 – 138.71 × Amide I, II ratio	0.38	26.039	< 0.01
Total metabolizable protein (MP) and degraded protein balance (DPB)^d^					
MP	Amide I, II ratio	Y = −110.83 – 142.07 × Amide I, II ratio	0.37	27.278	< 0.01
DPB	Amide I height, β-sheet height	Y = 22.61–1166.80 × Amide I height + 1508.67 × β-sheet height	0.74	13.358	< 0.01
Feed milk values based on metabolic characteristics of protein predicted by NRC system^e^					
FMV	Amide I, II ratio	Y = −1.62 + 2.45 × Amide I, II ratio	0.20	0.710	< 0.01

^a^ RSD, residual standard deviation

^b^MCP_RDP_, a microbial protein synthesized in the rumen based on available protein calculated as 0.85 of rumen-degraded protein; MCP_TDN_, a microbial protein synthesized in the rumen based on available energy (discounted TDN); AMCP, truly absorbed rumen-synthesized microbial protein in the small intestine

^c^ RUP, ruminally undegraded feed CP, calculated according to the formula in NRC–2001 dairy model; ARUP, truly absorbed rumen-undegraded feed protein in the small intestine

^d^ MP, metabolizable protein (a true protein that is digested postruminally and the component amino acid absorbed by the intestine); DPB, reflects the difference between the potential microbial protein synthesis based on ruminally degraded feed CP and that based on energy-TDN available for microbial fermentation in the rumen

^e^ FMV, feed milk value

## Discussion

Recently, the advanced vibrational spectroscopic techniques have been established to quantitatively estimate the primary and secondary molecular make-up of protein [26, 27]. Generally, the amide I and amide II bands are used to detect the information about protein concentration [28, 29]. However, the amide I is used more frequently than amide II to reveal the molecular structure of the protein, since the amide II originates from complex of vibrations that includes numerous functional groups such as ligneous compounds [30]. Our results showed that amide I area and peak height are the highest in the BPP7and BPP3 and lowest in the BPP2 and BPP6. These results are in agreement with the results obtained from the wet chemistry analysis [31]. The high CP or amide I area values are attributed to the high inclusion level of co-product (canola or carinata) in those BPPs. The total amide area was highly sensitive to the changes in blend BPPs composition, where the amide area increased with increasing the co-products levels or with decreasing pulse pea screenings levels in BPPs. For example, the BPP3 or BPP7 (carinata or canola meal 75% DM + pea screenings 25% DM) has a higher (*P* < 0.05) total amide area than BPP1 or BPP5 (carinata or canola meal 50% DM + pea screenings 50.0% DM).

The ratio of amide I to amide II has been found to be influenced by the heat-related processing of food/feed or by gene transformation of plant forage [32–34]. Previous studies noted that the amide I and II ratio had a positive correlation with the metabolizable protein [26]. Based on the current study results, the high amide I to amide II ratio of BPPs could be a consequence of the high inclusion level of co-products or adding co-product of carinata meal to BPPs. In agreement with these findings, Guevara-Oquendo et al. [31] reported a higher indigestible protein content in the BPP based on the co-product of canola meal (1.5% CP) than that of BPP based on the new co-product of carinata meal (3.2% CP). The low PC in BPP based on the new co-product of carinata meal is attributed to the greater content of NDICP and a lower content ADICP in the co-product of carinata meal compared with the co-product of canola meal. The NDICP is slowly degraded in the rumen and largely contributes to escaping protein from ruminal degradation [35]. Thus, a large amount of NDICP could reach the small intestine and hence, increasing the MP supply to dairy cows [35]. On the other hand, the ADICP reflects the amount of protein that is completely indigestible in the gastrointestinal tract [35]. Therefore, increasing the concentration of ADICP could limit the total tract digestibility of protein.

The secondary structures such as α-helix and β-sheet and their ratio are commonly used to detect the information about the protein’s molecular makeup [28, 36]. In the current study, all BPPs underwent the same processing, thus it is not surprising that the ratio was not changed among all BPPs. Yu and Samadi [37] found that the ratio of α-helix to β-sheet was altered by the moist heating of soybean and canola seeds. The alteration in the ratio of α-helix to β-sheet by thermal treatment was also reported by [38]. The changes in the secondary structure of the protein are possibly related to the denaturation of α-helix and β-sheet during thermal treatment. The current results showed that the ratio of α-helix to β-sheet was decreased with decreasing the level of co-products in the BPPs, which would reflect a reduction in the MP supply in BPPs. In agreement with these results, Guevara et al. [17] found that MP supply has been reduced by decreasing the inclusion level of the co-products of carinata or canola meal in BPPs.

**Fig. 2:**
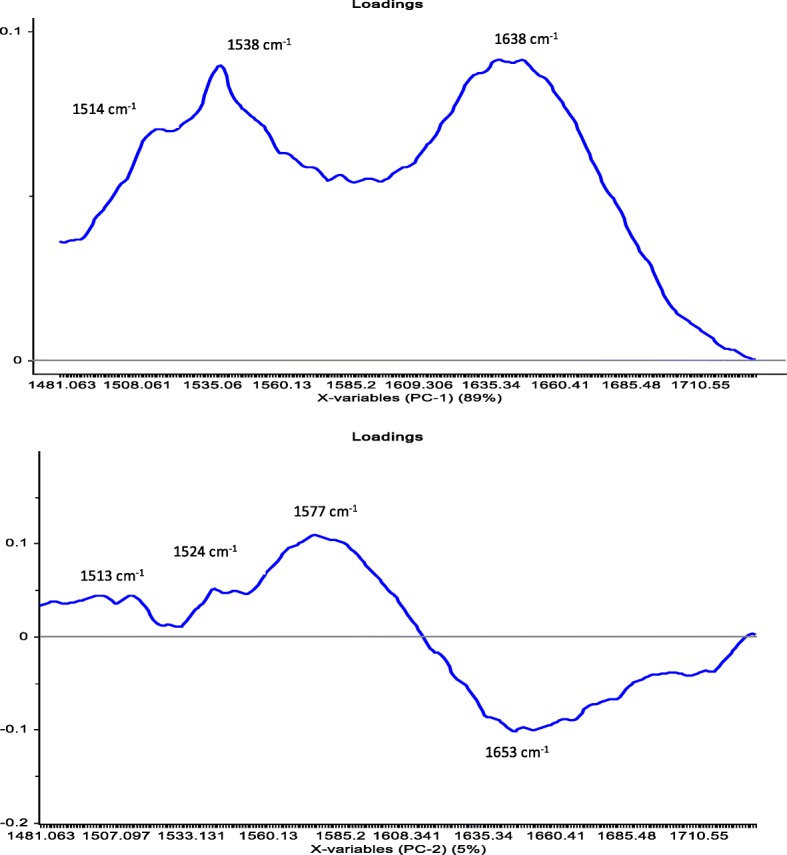
Loadings of the first two main components chosen based on the score plot of the preprocessed data (original spectra)

**Fig. 3:**
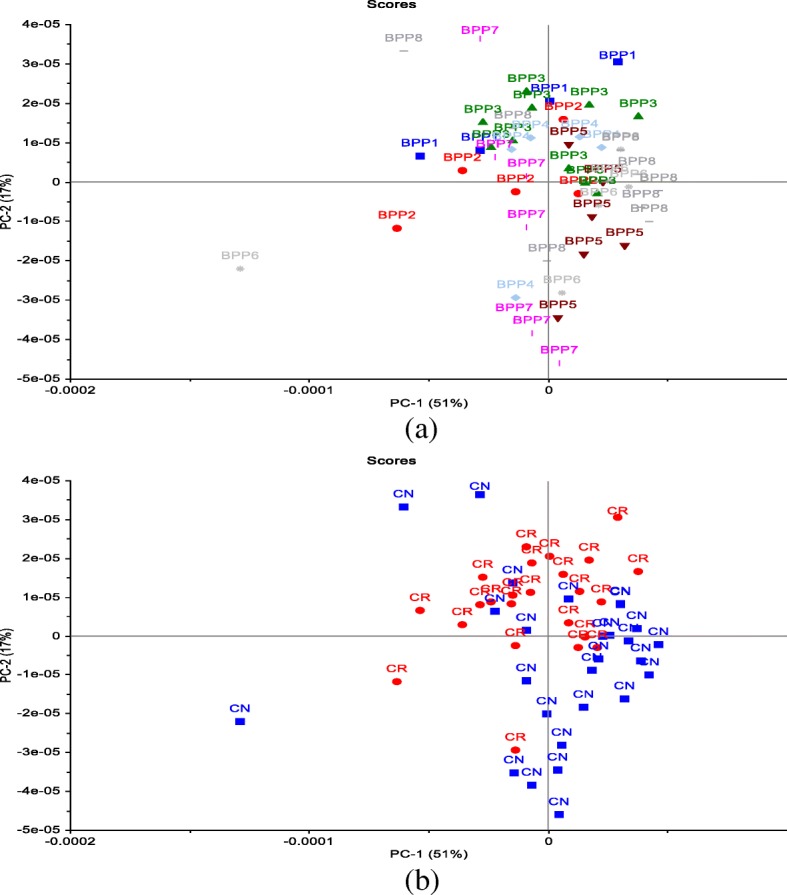
Two-dimensional score plot of the preprocessed (original) data represents grouping of spectra along PC1 and PC2 components, describing in total 94% of variability in the blend pelleted co-products: **a**) Effect of blend pelleted products (BPP) on the molecular structure changes related to protein region; **b**) Effect of co-products on the molecular structure changes related to protein region: carinata meal (CR) vs. canola meal (CN)

**Fig. 4:**
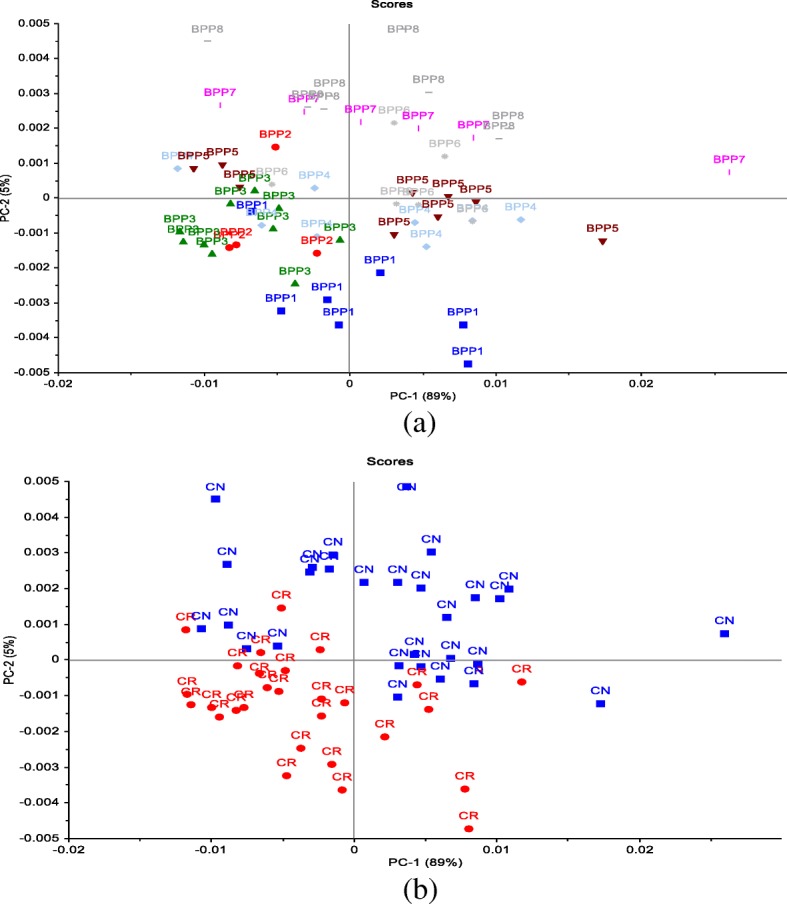
Two-dimensional score plot of the second derivative data represents grouping of spectra along PC1 and PC2 components, describing in total 68% of variability in the blend pelleted co-products: **a**) Effect of blend pelleted products (BPP) on the molecular structure changes related to protein region; **b**) Effect of co-products on the molecular structure changes related to protein region: carinata meal (CR) vs. canola meal (CN)

The PCA analysis in the current study was used to reduce the number of variables. The PCA was performed on the molecular structure related to protein region (ca. 1480–1730 cm^−1^). The first two PCs derived from the PCA classification of these spectra described 94% of the variance in the BPPs (Fig. 2a, b). Most of the BPPs based on the co-product of canola meal such as BPP6, BPP7 and BPP8 were clearly separated from the BPPs based on the new co-product of carinata meal by the PC2 which accounted for 5% of the variance. The BPP1 had exhibited the least negative values in PC2, while the BPP7 and BPP8 had the highest positive values. The PC1 which account for 89% of the variations among BPPs in terms of the molecular structure features did not cluster most of the BPPs. The overlapping between BPPs in the PC1 would indicate that these pellets had similar molecular structure features in the amide region. The loading point plots were used to determine the most important regions responsible for the clustering (Fig. 4). The amide I peak at ca. 1650 cm^−1^ was heavily loaded in PC1 and PC2, which separated the negative scores of spectra that belong to BPPs based on the new co-product of carinata meal from the positive score of the spectra that related to the BPP6, BPP7, and BPP8 (Fig. 3). These findings indicate that the amide region at ca. 1650 cm^−1^ of PC2 was the most important parameters for discriminating the BPPs. These data demonstrated that the amide I peak at ca. 1650 cm^−1^ for BPPs based on the new co-product of carinata meal was lower than that of the BPP6, BPP7, and BPP8. These data are in agreement with the univariate analysis (Table 2) that showed BPPs based on the co-product of canola meal were significantly higher in the amide I peak height compared with BPPs based on the new co-product of carinata meal. Based on these findings, the amide I band which is sensitive to small differentiation in molecular structure and hydrogen bonding motifs is important in the determination of protein structural and conformational changes.

**Fig. 5:**
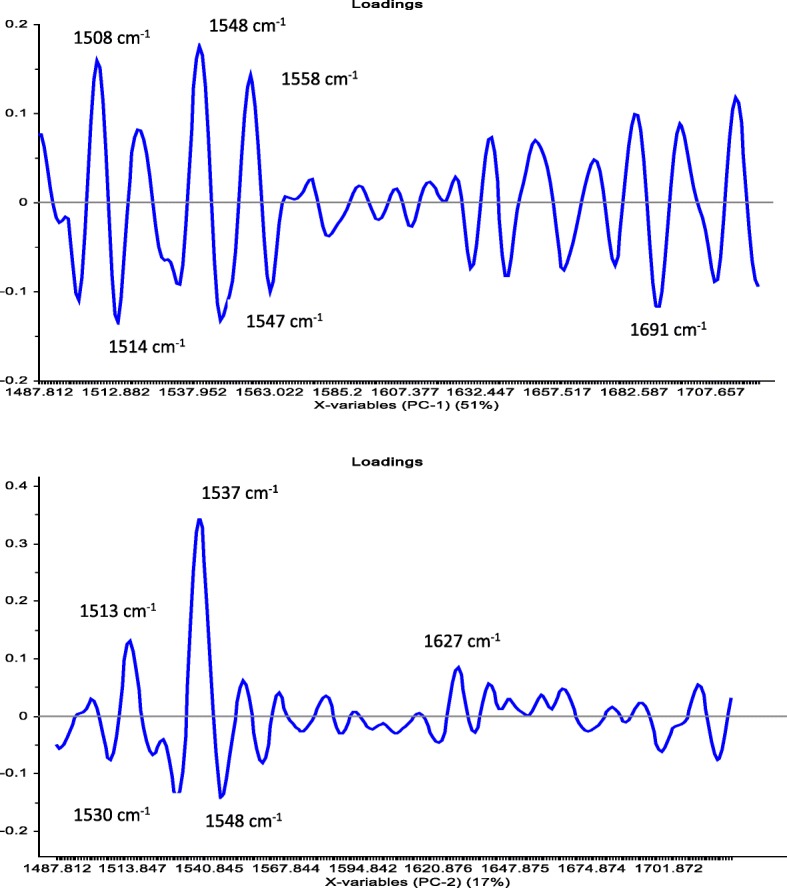
Loadings of the first two main components chosen based on the score plot of the second derivative data

In order to obtain clear and precise peak positions of protein bands by FT/IR-ATR, the raw spectra were processed by taking the second derivative (Fig. 4 a,b), which gives a negative peak for each band and shoulder in the absorption spectrum, and hence allows us to identify the individual peaks among complex spectra. The PCA score plot demonstrated that the clusters of all BPPs were overlapped along PC1 (51%) and PC2 (17%). The PCA loading plots of PC1 and PC2 are shown in Fig. 5. The loading plot showed that the variations along PC2 could be explained by the positive loading in the Amide II region (centered at ca. 1548 cm^−1^; N-H (60%) bending and C-N (40%) stretching vibrations: proteins α-helix), which separated the negative score of some BPPs based on the co-product of canola meal from the positive score of BPPs based on the new co-product of carinata meal.

The correlation analysis between the vibrational spectral features and protein profiles, protein subfractions and the predicted energy values of BPPs. Our results for the correlation between CP and primary structure and secondary structure are in agreement with previous studies that reported positive correlations of CP with amide I area and amide I height of the co-product of carinata meal or canola meal samples [39]. Furthermore, the correlation between NDICP and primary structure are similar to the previous studies that showed NDICP had a negative correlation with the amide II height and a positive correlation with the ratio of amide I to amide II height [36]. Our results showed there was no correlation (*P* > 0.05) between the metabolic and net energy values by the NRC-model and the molecular structure features related to protein region. In line with findings, [25] did not detect any association between the energy values and the molecular structure characteristics of the protein.

For the CNCPS fractions, the current study results are in agreement with [40] who reported a positive correlation between PB2 subfraction with the amide I to amide II height ratio. However, there was no association between the CNCPS fractions and α-helix to β-sheet ratio. In agreement with observations, Huang et al. [4] reported no correlation between α-helix to β-sheet ratio and the protein subfractions estimated by the CNCPS model.

For the *in situ* degradation kinetic parameters, a previous study noted that the ratio of amide I to amide II was highly correlated with the *in situ* protein degradation kinetic parameters [36]. These associations have been found to be affected by the enzymatic digestion of protein [36]. It has been observed the changes in the ratio of α-helix to β-sheet ratio would induce alterations in the protein molecular makeup [41]. The thermal treatment has been found to decrease the solubility of protein and increase the ADICP and NDICP as a consequence of protein denaturation during the heating process. Furthermore, the heat treatment could increase the cross-linkages among the amino acids in the polypeptide chain and reduce the sugars and finally decrease the solubility of CP [42]. Previous studies found that applying the heat treatment and increasing the heating time, caused an increase in the α-helix: β-sheet ratio in flaxseed and bio-ethanol co-products, respectively [34, 36]. In the current study, the α-helix to β-sheet ratio was correlated with the slowly degradable fraction of CP (*r* = − 0.44, *P* = 0.01) and the undegradable fraction of CP (*r* = 0.45, *P* = 0.01). These findings are in agreement with a previous study that found strong correlations between the α-helix to the β-sheet ratio of camelina seeds and the *in situ* protein degradation parameters [13]. The correlation coefficient values in this study were lower than that reported by Khan et al. [13] due to the diversity in protein origin in BPPs which applied adverse effects on the accuracy of predictions.

## Conclusions

In conclusion, the results in the current study indicated that the molecular structure features related to the protein of the blend pelleted products based on the co-product of canola or carinata meal could be revealed by the ATR-FT/IR spectroscopy. The univariate analysis showed differences in the absorption of the functional groups in the intestines related to the primary structure of the protein. The secondary structure of protein i.e. α-helix, β-sheet height ratio did not affect by BPP because all ingredients underwent to the same processing condition. The amide I to amide II height ratio was the best spectral parameter to estimate the changes in the protein degradation and the metabolizable protein of BPP.

## Additional file

Additional file 1: **Figure S1.** Typical FTIR spectra of blend pelleted products (BPP) based on carinata with pea screenings or canola meal with pea screenings. (PDF 182 kb)

## Data Availability

The datasets during and/or analyzed during the current study available from the corresponding author on reasonable request.

## Abbreviations

2D: Two-dimensional score plots
ADICP: Acid detergent insoluble crude protein
AECP: Truly absorbable endogenous protein in the small intestine
AMCP: Absorbable microbial protein
ARUP: Truly absorbable rumen undegraded feed protein
ATR-FT/IR: Fourier transform infrared spectroscopy with attenuated total reflectance
BCP: Bypass crude protein
BPP: Blend pelleted products
CNCPS: Cornell Net Carbohydrate and Protein System
CP: Crude protein
D: Potentially degradable fraction (%)
DE: Digestible energy
DE_3×_: Digestible energy at the production level of intake
DM: Dry matter
FMV: Feed milk value
Kd: Degradation rate (%/h)
Kp: Passage rate (%/h)
ME: Metabolizable energy
ME_3×_: Metabolizable energy at the production level of intake
MP: Metabolizable protein
NDICP: Neutral detergent insoluble crude protein
NE: Net energy
NEL_3×_: Net energy for lactation at the production level
NLIN: Nonlinear
NPN: Non-protein nitrogen
PC: Principal component
PCA: Principal Component Analysis
R(t): Residue present at t h incubation (%)
SCP: Soluble crude protein
T0: Lag time (h)
tdCP: Truly digestible crude protein
TDN: Total digestible nutrient
TDN_1×_: Total digestible nutrient at the maintenance level
U: Undegradable fraction (%)
