# Supplementary material for: Interactive association between processing induced molecular structure changes and nutrient delivery on a molecular basis, revealed by cutting-edge vibrational biomolecular spectroscopy
Source: J Anim Sci Biotechnol. 2019 Oct 22;10:85. doi: 10.1186/s40104-019-0384-z (PMC6805570; doi:10.1186/s40104-019-0384-z)
Supplement: Supplementary file 1 — Figure S1. Typical FTIR spectra of blend pelleted products (BPP) based on carinata with pea screenings or canola meal with pea screenings. (PDF 182 kb) [file 40104_2019_384_MOESM1_ESM.pdf]

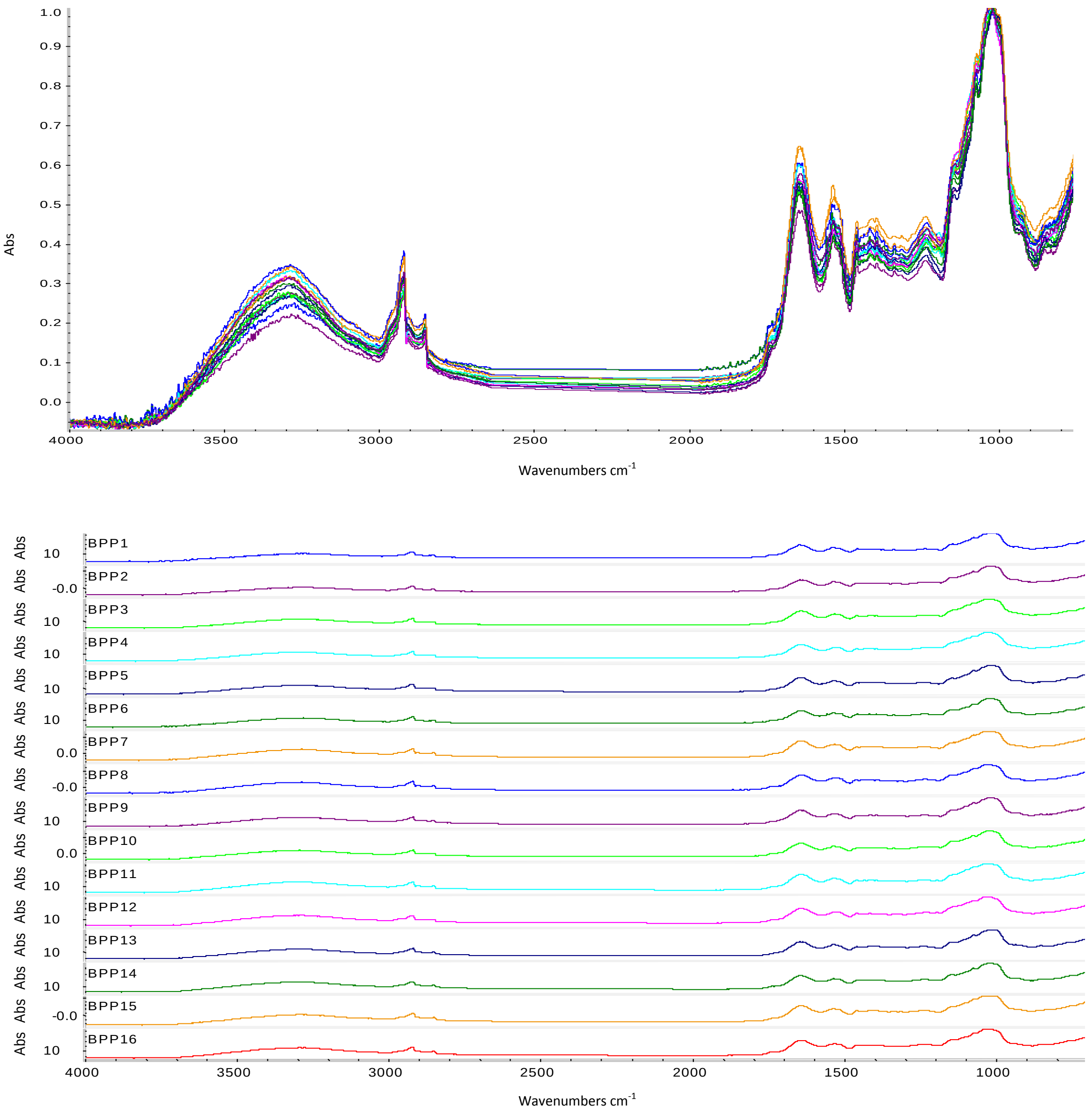

Fig1 S1. Typical FTIR spectra of blend pelleted products (BPP) based on carinata with pea screenings or canola meal with pea screenings.
